# O.2.2-4 Longitudinal associations between residential greenness exposure, physical activity and sedentary behavior levels: a country-wide study in Luxembourg

**DOI:** 10.1093/eurpub/ckad133.119

**Published:** 2023-09-11

**Authors:** Juliette Francisca Elain Van Beek, Camille Perchoux, Laurent Malisoux, Olivier Klein, Torsten Bohn, Mariëlle Beenackers, Frank van Lenthe, Martin Dijst

**Affiliations:** Luxembourg Institute of Socio-Economic Research, Esch-sur-Alzette, Luxembourg; Luxembourg Institute of Socio-Economic Research, Esch-sur-Alzette, Luxembourg; Luxembourg Institute of Health, Luxembourg, Luxembourg; juliette.vanbeek@liser.lu; Luxembourg Institute of Socio-Economic Research, Esch-sur-Alzette, Luxembourg; Luxembourg Institute of Health, Luxembourg, Luxembourg; juliette.vanbeek@liser.lu; Erasmus Medical Center, Rotterdam, The Netherlands; Erasmus Medical Center, Rotterdam, The Netherlands; Luxembourg Institute of Socio-Economic Research, Esch-sur-Alzette, Luxembourg

## Abstract

Greenness exposure has been associated with many health benefits, by providing opportunities for physical activity. Longitudinal studies are lacking, and most studies overlook the varying effects of different greenness types on physical activity (PA) and sedentary behavior (SB). We investigated 9-year associations of greenness characterized by overall greenness, vegetation type, and mix, with PA and SB using data from the ORISCAV-LUX study and the MET’HOOD project.

The PA and SB outcomes were measured using the International Physical Activity Questionnaire (IPAQ) short form. PA is expressed as MET-minutes/week and log-transformed, and SB is expressed as sitting time in minutes/day. The exposure variables were Tree Cover Density (TCD), Soil-adjusted Vegetation Index (SAVI), and Green Land Use Mix (GLUM). The study population consisted of 628 adults, who completed the questionnaires between January 2007-2009 and January 2016-July 2017. Missing data were imputed. Greenness exposure was collected within 800-meter street network buffers based on participants’ exact residential address. Associations between TCD, SAVI and GLUM and PA and SB were estimated using a Random Effects Within-Between (REWB) model. Interaction by gender and SES were explored. Sensitivity analyses were performed on smaller (500 meter buffer) and larger (1000, 2000 meter buffers) definitions of the residential neighborhood.

We found evidence of between-individual associations of GLUM and PA (β = 0.17, 95% CI: 0.03; 0.91), and within-individual associations of TCD with PA (β = 0.03, 95% CI: 0.002; 0.438) (in 800 meter buffers). There was no evidence for associations between greenness exposure and SB. Sensitivity analysis using varying buffer sizes showed similar associations.

Our study found evidence of associations of GLUM and TCD with PA, and no associations between greenness exposure and SB. Promoting different types of greenness may result in different PA levels and behaviors. Replication studies are needed to inform urban planning interventions.

This study is funded by the EU Horizon2020 Program under grant agreement number 956780 (SURREAL). The ORISCAV-LUX study is funded by the Luxembourg Ministry of Health and Ministry of Higher Education and Research. The MET'HOOD project is funded by the Luxembourg National Research Fund as part of the CORE 2020 programme (C20/BM/14787166).

